# Mutation of regulatory phosphorylation sites in PFKFB2 worsens renal fibrosis

**DOI:** 10.1038/s41598-020-71475-z

**Published:** 2020-09-03

**Authors:** Mardiana Lee, Geoff Harley, Marina Katerelos, Kurt Gleich, Mitchell A. Sullivan, Adrienne Laskowski, Melinda Coughlan, Scott A. Fraser, Peter F. Mount, David A. Power

**Affiliations:** 1 Kidney Laboratory, Department of Nephrology, Austin Health, Heidelberg, VIC 3084 Australia; 2 Department of Medicine, The University of Melbourne, Heidelberg, VIC Australia; 3 Mater Research Institute-the University of Queensland, Translational Research Institute, Woolloongabba, QLD Australia; 4 Glycation, Nutrition and Metabolism Laboratory, Department of Diabetes, Central Clinical School, Monash University, Melbourne, VIC Australia; 5 The Institute for Breathing and Sleep (IBAS), Austin Health, Heidelberg, VIC Australia

**Keywords:** Biochemistry, Diseases, Nephrology

## Abstract

Fatty acid oxidation is the major energy pathway used by the kidney, although glycolysis becomes more important in the low oxygen environment of the medulla. Fatty acid oxidation appears to be reduced in renal fibrosis, and drugs that reverse this improve fibrosis. Expression of glycolytic genes is more variable, but some studies have shown that inhibiting glycolysis reduces renal fibrosis. To address the role of glycolysis in renal fibrosis, we have used a genetic approach. The crucial control point in the rate of glycolysis is 6-phosphofructo-2-kinase/fructose-2,6-biphosphatase. Phosphorylation of the kidney isoform, PFKFB2, on residues Ser^468^ and Ser^485^ stimulates glycolysis and is the most important mechanism regulating glycolysis. We generated transgenic mice with inactivating mutations of Ser^468^ and Ser^485^ in PFKFB2 (PFKFB2 KI mice). These mutations were associated with a reduced ability to increase glycolysis in primary cultures of renal tubular cells from PFKFB2 KI mice compared to WT cells. This was associated in PFKFB2 KI mice with increased renal fibrosis, which was more severe in the unilaternal ureteric obstruction (UUO) model compared with the folic acid nephropathy (FAN) model. These studies show that phosphorylation of PFKFB2 is important in limiting renal fibrosis after injury, indicating that the ability to regulate and maintain adequate glycolysis in the kidney is crucial for renal homeostasis. The changes were most marked in the UUO model, probably reflecting a greater effect on distal renal tubules and the greater importance of glycolysis in the distal nephron.

## Introduction

**Figure 1:**
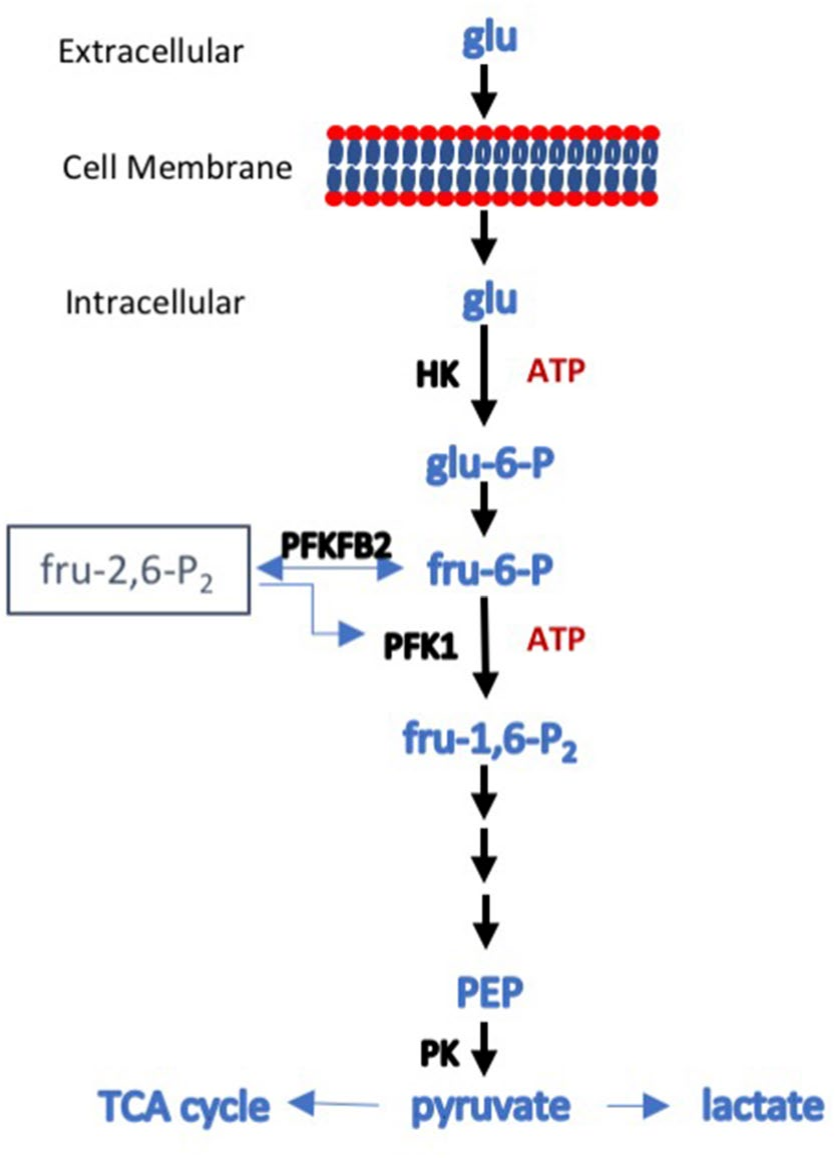
Schematic diagram of glycolysis. Glucose enters the cell via one of the glucose transporters and is phosphorylated, in an ATP-consuming step catalyzed in most cells by hexokinase (HK), to glucose-6-phosphate that cannot exit the cell. An isomerase converts it to fructose-6-phosphate, which is then converted to fructose-1,6-bisphosphate in a second ATP-consuming step catalyzed by phosphofructokinase 1 (PFK1). PFK1 catalysis is the major rate-limiting step in glycolysis and the first committed step. The activity of PFK1 is regulated by a number of factors, but one of the most important is the level of fructose-2,6-bisphosphate. Synthesis and degradation of fru-2,6-P_2_ is under the control of 6-phosphofructo-2-kinase/fructose-2,6-bisphosphatase (PFKFB), a bifunctional enzyme that catalyses both the formation and degradation of fru-2,6-P_2_. PFKFB exists as four isoforms, the products of separate genes, each with a distinct activity and tissue distribution. PFKFB2 has been described as the kidney isoform, as it is well expressed. Phosphorylation of PFKFB2 on either Serine^366^ and Serine^382^ increases formation of fru-2,6-P_2_ and increases the enzymatic activity of PFK1, driving glycolysis forward. The fate of fru-1,6-P_2_ is shown in outline. Fru-1,6-P_2_ is unstable and is converted to two 3-carbon sugars that eventually end as two molecules of phosphoenolpyruvate (PEP). This is then converted to pyruvate by pyruvate kinase (PK), which can then be converted to lactate, usually under anaerobic conditions, or enter the tricarboxylic acid (TCA) cycle in mitochondria, generally when there is oxygen available. In the diagram, only three glycolytic enzymes (HK, PFK1, PK) are shown as these are the rate limiting steps and the most susceptible to regulation.

Recent evidence indicates that the development of renal fibrosis is accompanied by a reduction in fatty acid oxidation by tubular epithelial cells^1–3^. Drugs that reverse this process reduce fibrosis in experimental models^1, 4^. Interestingly, expression of some glycolytic pathway genes is also reduced in human renal fibrosis and in mouse models^1^. The exceptions are hexokinase and pyruvate kinase, which are at the start and end of the glycolytic pathway (Fig. 1)^1, 5^. Inhibition of hexokinase with 2-deoxyglucose, pyruvate kinase M2 (PKM2) with either the drug shikonen or RNAi, and pyruvate kinase with dichloroacetate reduces renal fibrosis in the UUO model^5, 6^. Inhibition of PKM2 also reduces injury in ischaemic AKI^7^.

While proximal convoluted tubules have a substrate preference for fatty acid oxidation and little glycolytic capacity, distal tubules often utilise glucose as their preferred energy source^8, 9^. In the inner medulla, for example, cells of the collecting duct utilise glycolysis predominantly^9, 10^. The thick ascending limb has a very high energy requirement and a substrate preference for glucose, which can then undergo either mitochondrial oxidative phosphorylation or anaerobic glycolysis^9, 11^.

The rate of glycolysis in cells is determined by the rate of glucose entry and the activity of three rate-limiting glycolytic enzymes, namely hexokinase, phosphofructokinase 1 (PFK-1) and pyruvate kinase^12^. Not surprisingly, activity of these enzymes is greatest in the distal nephron^9, 13^. PFK-1 converts glucose-6-phosphate to fructose-1,6-bisphosphate, and is the most important rate-limiting step in glycolysis and the first committed step^12^. PFK-1 is inhibited by phosphoenolpyruvic acid, a glycolytic intermediate, low pH, citrate, and ATP. It is activated by AMP and, most importantly, fructose-2,6-bisphosphate (F-2,6-P_2_)^14^. PFK-2/FBPase-2 (6-phosphofructo-2 kinase/fructose-2,6-bisphosphatase) plays a key role in the regulation of glycolysis, by catalysing both the synthesis and degradation of fructose 2,6-bisphosphate (Fru-2,6-P_2_)^12^. There are four isoforms of PFK-2/FBPase-2, designated PFKFB1-4^15^. The activity of PFKFB3 and PFKFB4 have been most strongly associated with aerobic glycolysis in tumours, where glycolysis proceeds to lactate rather than mitochondrial oxidative phosphorylation, despite the presence of oxygen^16^. The role of PFKFB2 in regulating cellular metabolism is shown in Fig. 1.

In the kidney, PFKFB2 is more strongly expressed than the other isoforms^17^, and has sometimes been described as the kidney isoform^15^. PFKFB2 is also the dominant isoform in the heart and its regulation by phosphorylation is well described^15^. A number of stimuli, including adrenaline, insulin, anoxia, and workload lead to phosphorylation of PFKFB2 in the heart, thereby leading to an increase in Fru-2,6-P_2_ concentration and increased glycolysis^15^. These effects are mediated by phosphorylation of PFKFB2 in its C-terminal domain by protein kinases including protein kinase A (PKA), Akt and AMPK. The two main activating phosphorylation sites identified are murine Serine 468 (Serine 466 in the human; a target for PKA, Akt and AMPK), and Serine 485 (Serine 483 in the human; a target for PKA and Akt)^15^. The sites are referred to throughout using the murine numbering to reduce confusion.

To determine the role of regulation of tubular glycolysis by PFKFB2 in the development of renal fibrosis, we developed a transgenic mouse that has inactivating mutations of the regulatory Ser^468^ and Ser^485^ phosphosites in PFKFB2. The effect of these mutations was predicted to be a reduced ability to stimulate glycolysis.

## Methods

### Generation of PFKFB2 KI mice

Mice with inactivating mutations of the phosphorylation sites Ser^468^ and Ser^485^ in PFKFB2 (PFKFB2 KI mice) were generated on a C57Bl/6 background by OzGene Pty Ltd, Bentley DC, WA, Australia. Briefly the phospho-acceptor sites Ser^468^ and Ser^485^ located in exon 15 of the mouse PFK2 gene were mutated to Alanine (strategy shown in Supplementary Fig. 1). A wild type copy of exon 15 encased by lox P sites was inserted into intron 4–5 upstream of the exon 15 splice acceptor followed by a polyadenylation signal. A neomycin selection cassette was targeted into intron 14–15 flanked by FRT sites. FlpE-mediated recombination was used to excise the neo cassette, leaving an FRT site in its place. Cre-mediated recombination through breading with Cre-deletor mice deleted the wild-type exon 15 sequences resulting in the expression of the mutant PFK2 protein at normal levels. Mice were bred as PFKFB2 KI heterozygotes. Mice homozygous for the PFKFB2 KI mutation and mice with homozygous WT PFKFB2 were generated by brother-sister mating. Mouse genotypes were confirmed by PCR.

### Animal studies

All experiments were approved following submissions to the Austin Health Animal Ethics Committee. This Committee is constituted by Austin Health, a public hospital supported and monitored by the Victorian State Government, under guidelines prepared by the National Health and Medical Research Council (NHMRC) of Australia, the Commonwealth Scientific and Industrial Research Organisation (CSIRO), the Australian University Vice-Chancellor’s Committee, and Animal Welfare Victoria. The unilateral ureteric obstruction (UUO) model and the folic acid nephropathy (FAN) model were used to create renal fibrosis, as we have recently described^4^.

### Cell culture

Fibroblasts were isolated from WT and PFKFB2KI mouse kidneys using a modified method^18^.

Briefly, kidneys were removed from euthanized mice, and chopped into small pieces using a scalpel blade. Kidneys were then placed in 10 ml collagenase-dispase medium (ThermoFisher Scientific) and incubated at 37 °C for 45 min with constant shaking. Fibroblast medium (DMEM/F12; 15% FBS; 12.5 mM Hepes, Pen/Strep) was added to the digest to inactivate the enzymes and the samples centrifuged at 500×*g* for 5 min. The supernatant was removed and 20 ml fresh fibroblast medium added and the sample centrifuged as described above. This was repeated two more times and then the pellet was resuspended in 20 ml fibroblast medium and transferred to two 10 cm tissue culture dishes. The plates were checked daily and the media changed as required. Fibroblasts usually exit tissue fragments within 2–5 days. When there were sufficient patches of confluent cells, the cells were trypsinized and plated into 6-well plates. Once confluent, cell lysates were prepared.

Primary cultures of renal tubular epithelial cells (TEC) were prepared by sieving whole mouse kidneys, as we have recently reported^4^. These cultures are likely to contain cells of a variety of tubular lineages.

### Cell metabolism assays

The glycolytic and mitochondrial function of TEC from wild type and PFKFB2 KI mice was measured using the XFe96 Seahorse analyser and XFe96-FluxPaks containing 96-well plates and cartridges (Agilent Technologies, Santa Clara, CA, USA). Confluent cells were dissociated from the plate using 0.25% Trypsin-EDTA), resuspended in KI media and counted using the Countess II Cell Counter (Thermo Fischer Scientific, Massachusetts, USA). 80,000 cells in 180 μl were added to each well in a Seahorse XF96 cell culture microplate in growth medium (DMEM/F12 supplemented with 5% FCS, ITS, 25 mM HEPES, 100 U/ml penicillin, 100 mg/ml streptomycin, 1 ng/ml prostaglandin, 5 × 10^–11^ M triiodothyronine, 5 × 10^–8^ M hydrocortisone, and 25 ng/ml mouse epidermal growth factor) and allowed to settle for at least 24 h. One day prior to measurement, the sensor cartridge was hydrated in Seahorse XF Calibrant Solution overnight at 37 °C in a non-CO_2_ incubator. One hour prior to the experiment the cells were washed 3 times and then incubated in Seahorse XF Base Medium. This was supplemented with 2 mM glutamine, pH 7.4 for the glycolytic stress test and with 10 mM glucose and 1 mM pyruvate in addition to glutamine for the mitochondrial stress test. Glycolytic and mitochondrial function were analyzed according to Seahorse XF Glycolysis Stress Test and Seahorse XF Mito Stress Test protocols (Agilent Technologies, Santa Clara, CA, USA).

### Western blot analysis

Kidney lysates were prepared and Western blots performed as previously described^19^. Quantification of Western Blots was by densitometry with analysis using Image J software^20^. The following antibodies were used in the Western Blot analysis: anti-α-Smooth Muscle Actin—FITC antibody (Sigma-Aldrich, St. Louis, USA), anti-Fluorescein-POD Fab fragments (from goat Roche Applied Science, Indianapolis, USA), anti-GAPDH antibody (Rabbit monoclonal antibody, Cell Signaling technology, Massachusetts, USA), anti-Fibronectin antibody (Rabbit monoclonal antibody, Sigma-Aldrich, St.Louis, USA), anti-phospho-PFKFB2 (Ser^485^) (Rabbit monoclonal antibody, Cell Signaling Technology, Massachusetts, USA), anti-PFKFB2 antibody (Rabbit polyclonal antibody, abcam, Cambridge, UK), anti-PKM2 antibody (Rabbit monoclonal, Cell Signaling technology, Massachusetts, USA), anti-pPKM2 antibody (Rabbit polyclonal, Cell Signaling technology, Massachusetts, USA) and anti-rabbit Immunoglobulin HRP-linked (Swine polyclonal, Dako, Agilent Pathology Solutions, Santa Clara, USA).

### Histology

Analysis of renal fibrosis by Picro-Sirius Red staining was performed as previously described^4^. Kidneys were sliced in half transversely and fixed in formalin. Kidney sections were stained using Picro-Sirius Red Stain Kit (Abcam, Cambridge, UK) according to the manufacturer’s instructions. Samples were processed by the Department of Anatomical Pathology, Austin Health. The area occupied by collagen in Picro-Sirius Red stained sections was measured using Image J software. The values obtained were expressed as a percentage of the whole cortical area.

### Real-time polymerase chain reaction (qRT-PCR)

Total RNA was purified from whole mouse kidney samples, or cell cultures and reverse transcribed as previously described^19^. Primer efficiency was measured using standard dilution, and the Pfaffl method was used to calculate relative expression^21^. Data was expressed as fold expression relative to littermate WT controls. Primer sequences are shown in Supplementary Table 1.

### Glycogen assay

Glycogen was extracted and quantified using a procedure similar to that previously described^22^. Briefly, approximately 100 mg of frozen kidney tissue was lyophilized, ground and boiled in 300 μL of 30% [w/v] KOH for 1 h, followed by ethanol precipitation (80% [v/v], 15 mM LiCl) for a minimum of 1 h at − 30 °C. Ethanol precipitation was repeated three more times, with the final pellet being re-dissolved in water. Using a method similar to that performed previously, glycogen was degraded to glucose using amyloglucosidase^23^. Using an established glucose determination assay^24^, 30 μL of degraded glycogen and 30 μL of d-glucose standards up to 400 μg/mL was mixed with 170 μL of reaction buffer containing: 150 μL of 200 mM tricine/KOH (pH 8) and 10 mM MgCl_2_, 18 μL of deionized water, 1 μL of 112.5 mM NAPD, 1 μL of 180 mM adenosine triphosphate (ATP) and 0.5 U of glucose-6-phosphate dehydrogenase (G6PDH; Roche). The absorbance at 340 nm was initially recorded for 30 min, once per min, to determine a baseline, followed by the addition of 4 μL hexokinase solution (0.75 U in 5 μL of 200 mM tricine/KOH [pH 8] and 10 mM MgCl_2_) to each well. The absorbance at 340 nm was the recorded for 30 min, once per min. The concentration of glucose in each well was calculated by subtracting the baseline absorbance from the absorbance plateau, using the D-glucose standard curve. The average glucose concentration of the two water controls was subtracted from each sample, giving a final glucose concentration, which was used to calculate the glycogen content in the initial sample.

### Statistics

Statistics were performed using Prism version 7.0a for Mac OS X (GraphPad Software, San Diego, CA), as we have previously described^4^. Data are presented as means ± SD. RT-PCR and Seahorse data is presented as standard error of the mean. Multiple group means were compared by ANOVA followed by a post-hoc test. Comparison of means from two groups was performed by an unpaired t-test. P values of < 0.05 were considered significant.

## Results

### Generation and characterisation of PFKFB2 KI mice

PFKFB2 KI mice were maintained as a homozygous line on a C57BL/6 background. Wild type (WT) mice were derived from heterozygous matings of PFKFB2 KI mice and maintained as a separate line. Comparing the two lines, there was no difference in plasma glucose, but the kidneys were smaller and plasma urea was also significantly less (Table 1). Histologically, there was no abnormality in the PFKFB2 KI kidneys (data not shown).

**Table 1:** Comparative physical and biochemical data from PFKFB2 KI mice (n = 9–10).

	WT	PFKFB2 KI	P-value
Body weight (g)	25.41 ± 2.61	23.80 ± 1.42	0.356
Right kidney (mg)	212.60 ± 24.52	187.79 ± 17.66	0.023
Left kidney (mg)	214.40 ± 24.76	180.40 ± 16.44	0.003
Heart (mg)	195.60 ± 57.21	169.29 ± 31.42	0.239
Kidney (mg)/body weight (g)	8.48 ± 1.23	7.53 ± 0.62	0.005
Plasma glucose (mmol/L)	16.20 ± 3.23	16.01 ± 4.05	0.909
Plasma urea (mmol/L)	8.75 ± 1.70	7.22 ± 1.30	0.036

### Measurement of glycolysis in tubular epithelial cells (TEC)

**Figure 2:**
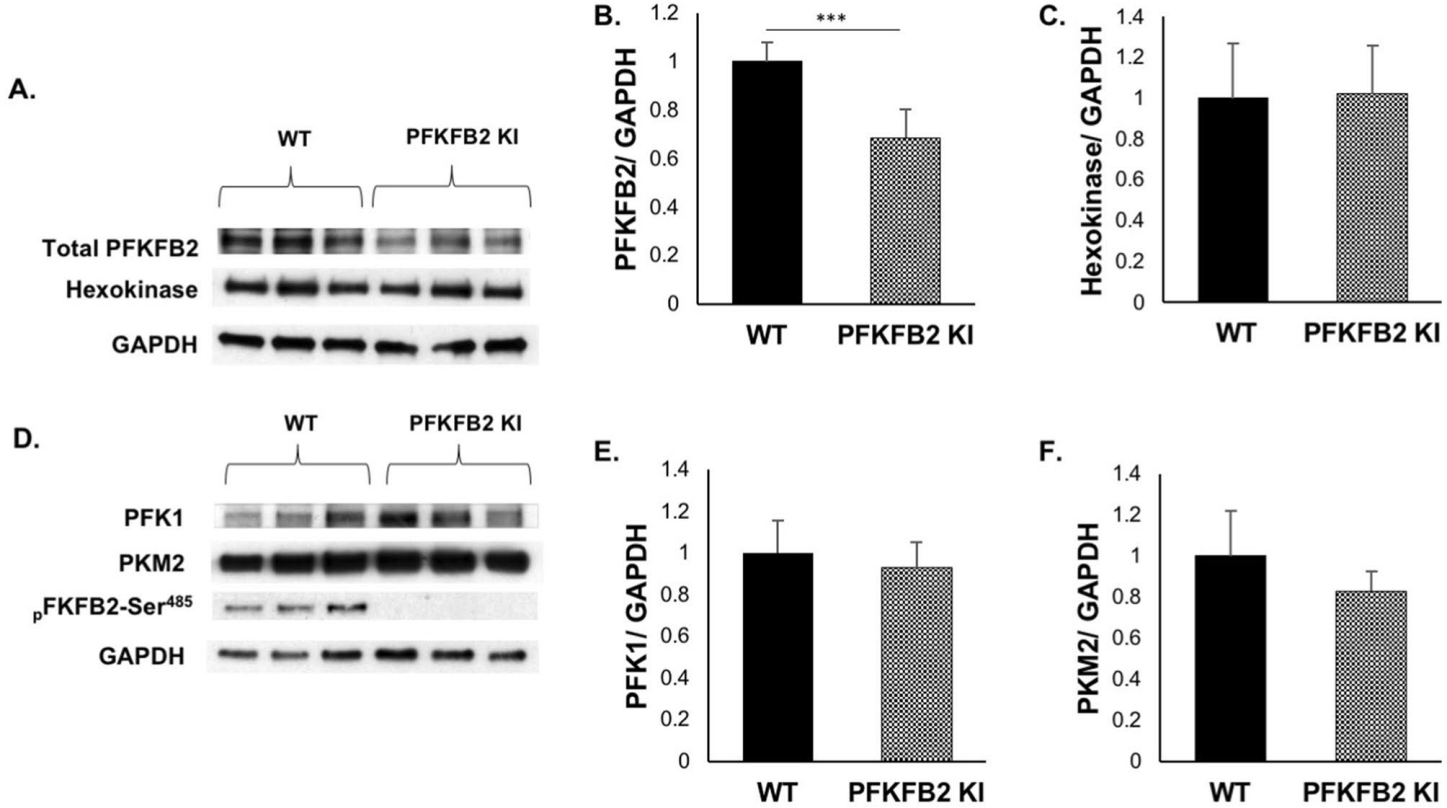
Expression of glycolytic pathway proteins in TEC from WT and PFKFB2 KI mice. (**A**–**F**) Primary cultures of tubular epithelial cells (TEC) and fibroblasts (Fib) were established and cell lysates prepared. Western blots showed that expression of the major proteins involved in glycolysis were unchanged in the cells from PFKFB2 KI mice compared to WT. Protein loading was determined by GAPDH. Expression of PFKFB2 was reduced in the PFKFB2 KI cells. ***p < 0.001. Mean + S.D.

To determine whether inactivating mutation of the Ser^468^ and Ser^485^ sites in PFKFB2 affected the ability of cells to increase glycolysis in response to stimuli, primary cultures of renal tubular epithelial cells were isolated from PFKFB2 KI mice (PFKFB2 KI TEC) and WT mice (WT TEC). Expression of the proteins that determine the rate of glycolysis, namely hexokinase, phosphofructokinase 1 (PFK1) and pyruvate kinase (PKM2) was unchanged in the PFKFB2 KI TEC compared to WT (Fig. 2A–F). There was an approximately 30% reduction in expression of PFKFB2 in the PFKFB2 KI TEC (Fig. 2B).

**Figure 3:**
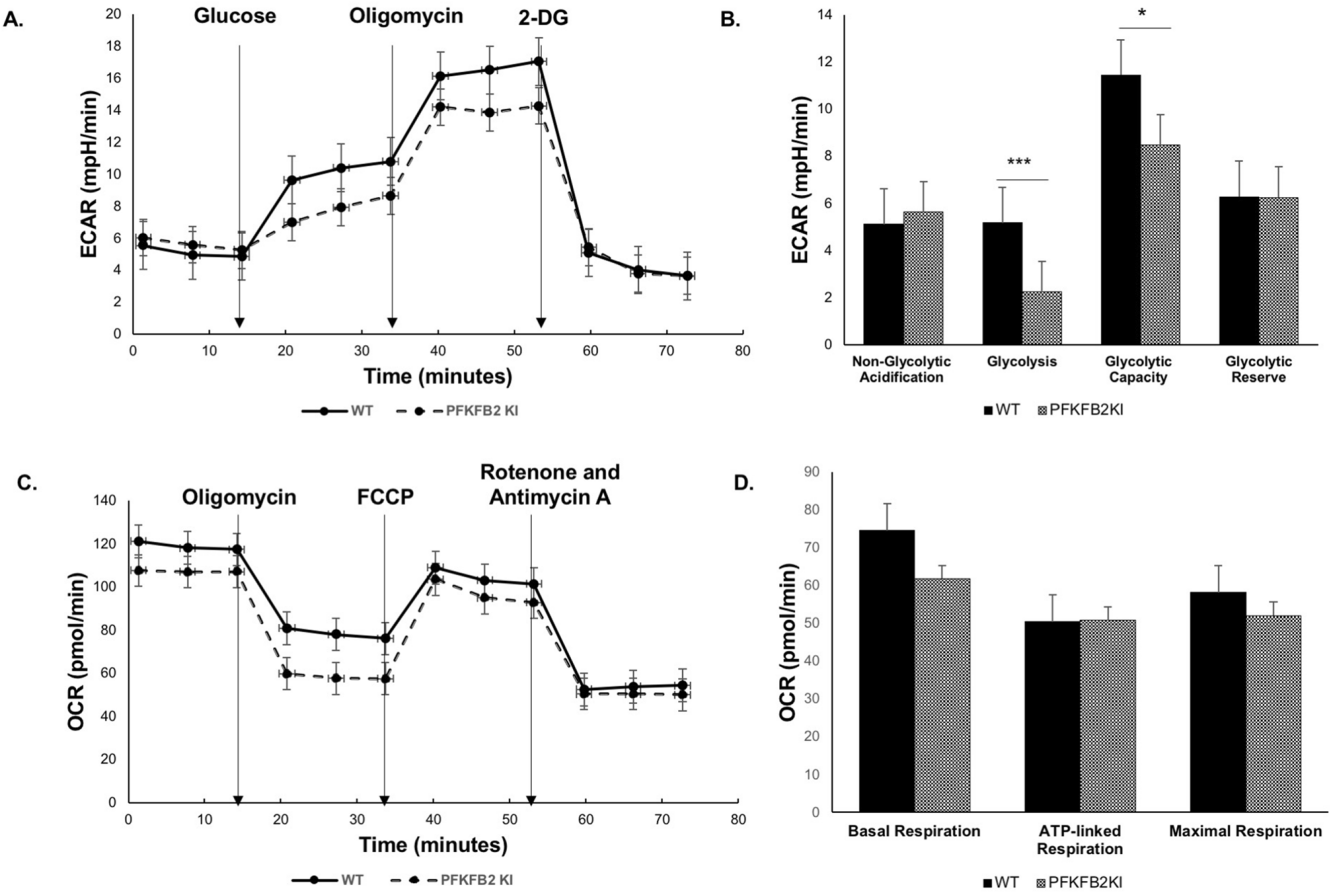
The glycolytic and mitochondrial function of mouse kidney epithelial cells from wild type and PFKFB2 KI mice was measured using the XFe96 Seahorse analyser. (**A**,**B**) There was reduced glycolysis in glucose-stimulated cells and reduced glycolytic capacity in PFKFB2 KI TEC compared to WT. (**C**, **D**) Mitochondrial respiration was not significantly different. *p < 0.05, ***p < 0.001. Mean + S.E.M.

The cells were studied in a Seahorse analyzer, using reagents in the Agilent Seahorse XF Glycolysis Stress Test Kit and the Mito Stress Test. There was significantly less glycolysis in the PFKFB2 KI TEC compared with WT TEC when incubated with glucose (Fig. 3A,B). Glycolytic capacity, which is a measure of the maximum rate of conversion to pyruvate or lactate that the cells can deliver, was also less in the PFKFB2 KI TEC but the difference was completely accounted for by reduced baseline glycolysis, rather than a difference in glycolytic reserve. The data indicate that other mechanisms are still able to regulate glycolysis, especially when the cells are stressed by injection of oligomycin, which inhibits ATP synthase and thus oxidative phosphorylation. There was no significant difference in mitochondrial respiration although there was a trend to a reduction in basal respiration in the PFKFB2 KI cells (Fig. 3C,D).

### PFKFB2 localization, expression and phosphorylation in disease models

**Figure 4:**
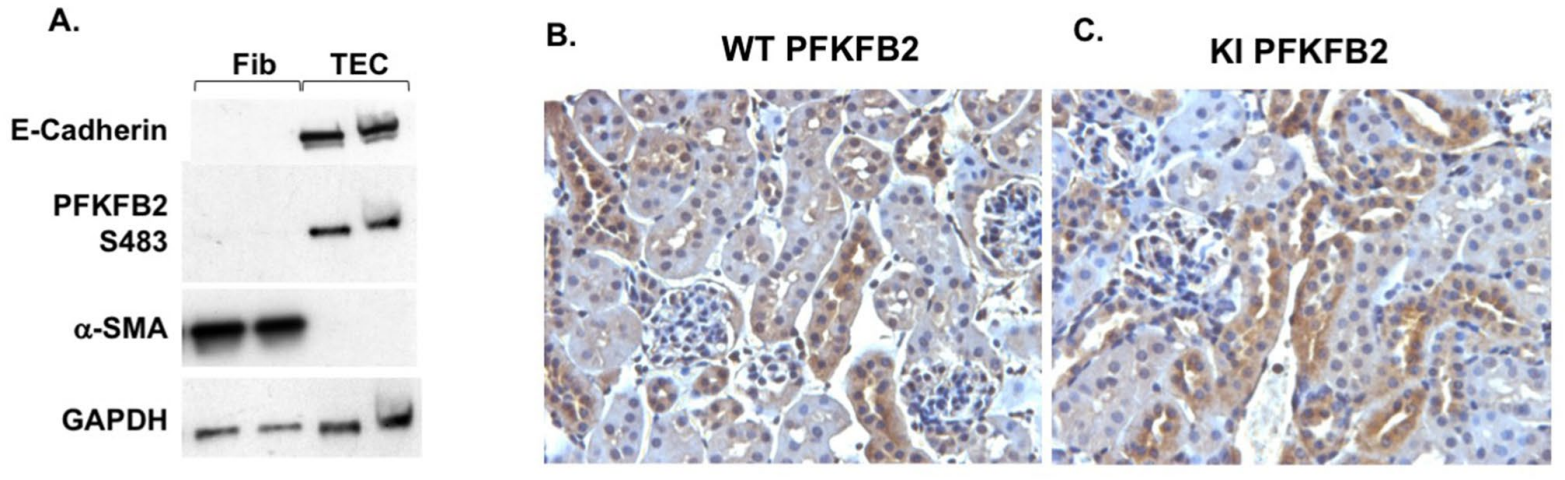
PFKFB2 and pPFKFB2 expression in TEC and fibroblasts from WT mice. (**A**) Primary cultures of tubular epithelial cells (TEC) and fibroblasts (Fib) were established and cell lysates prepared. Western blots showed that fibroblasts expressed α-smooth muscle actin (α-SMA) while TEC were expressed the epithelial marker E-cadherin. Protein loading was determined by GAPDH. Only the TEC were positive for PFKFB2-Ser^485^. (**B**,**C**) Immunohistochemical staining with anti-PFKFB2 Ab of kidneys from wild type (WT) and PFKFB2 KI mice. Staining is identical in both with predominant expression in tubules of the distal nephron.

To determine whether PFKFB2 was expressed in fibroblasts, tissue explants were used to derive primary cultures from WT mouse kidneys. Western blots showed that these cells expressed α-SMA but not the epithelial cell marker E-cadherin (Fig. 4A). They also did not react with anti-PFKFB2-Ser^485^ antibodies. In contrast, TEC reacted with anti-E-cadherin and anti-PFKFB2-Ser^485^ antibodies. Immunohistochemical staining with anti-PFKFB2 Ab showed that expression of PFKFB2 in normal kidneys was tubular, particularly affecting distal tubules (Fig. 4B). Immunohistochemical staining in kidneys from PFKFB2 KI mice showed an identical distribution to WT mice (Fig. 4C).

**Figure 5:**
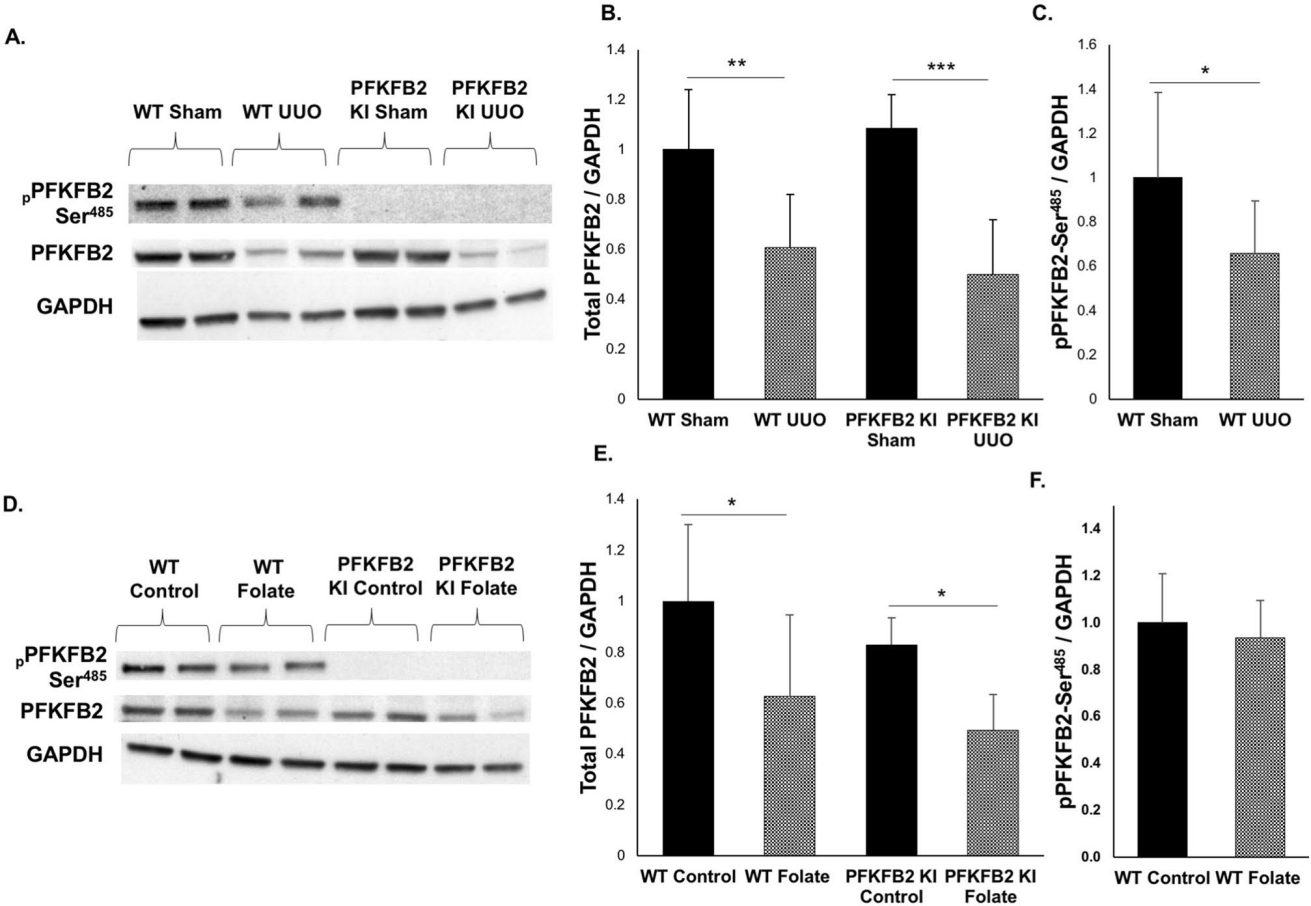
PFKFB2 and pPFKFB2 expression in WT mice and PFKFB2 KI mice with UUO and FAN. (**A**,**B**) Representative Western blots showing significantly reduced expression of PFKFB2 in WT and PFKFB2 KI mice with UUO, as determined by densitometry. There was, however, no difference between WT and PFKFB2 KI mice. (**C**) pPFKFB2-Ser^485^ was reduced in WT mice with UUO when normalized to GAPDH, although consistent with the reduced level of expression of PFKFB2. (**D**,**E**) Representative Western blots showing significantly reduced expression of PFKFB2 in WT and PFKFB2 KI mice with FAN, as determined by densitometry. Again, there was no difference between WT and PFKFB2 KI mice. (**F**) pPFKFB2-Ser^485^ was unchanged in WT mice with FAN when normalized to GAPDH. n = 6 mice per panel. *P < 0.05, **P < 0.01, ***P < 0.001. Mean + S.D.

To determine the level of expression of PFKFB2 and PFKFB2-Ser^485^ in disease, tissue lysates from control and fibrotic kidneys were probed with antibodies against PFKFB2 and pSer^485^ (Fig. 5A,D). In contrast to TEC isolated from PFKFB2 KI mice, there was no difference in expression of PFKFB2 between kidneys from WT and PFKFB2 KI control mice in either the UUO or FAN models (Fig. 5B,E). In both the UUO and FAN models, expression of PFKFB2 was reduced in WT and PFKFB2 KI mice to a similar degree. Phosphorylation of PFKFB2 at Ser^485^ was reduced in WT mice when normalized to GAPDH in the UUO model, but not the FAN model (Fig. 5C,F). Western blots using antibodies against the Ser^468^ phosphosite remained positive in the PFKFB2 KI due to the known homology with a similar site in PFKFB3, so the data is not shown^25^.

**Figure 6:**
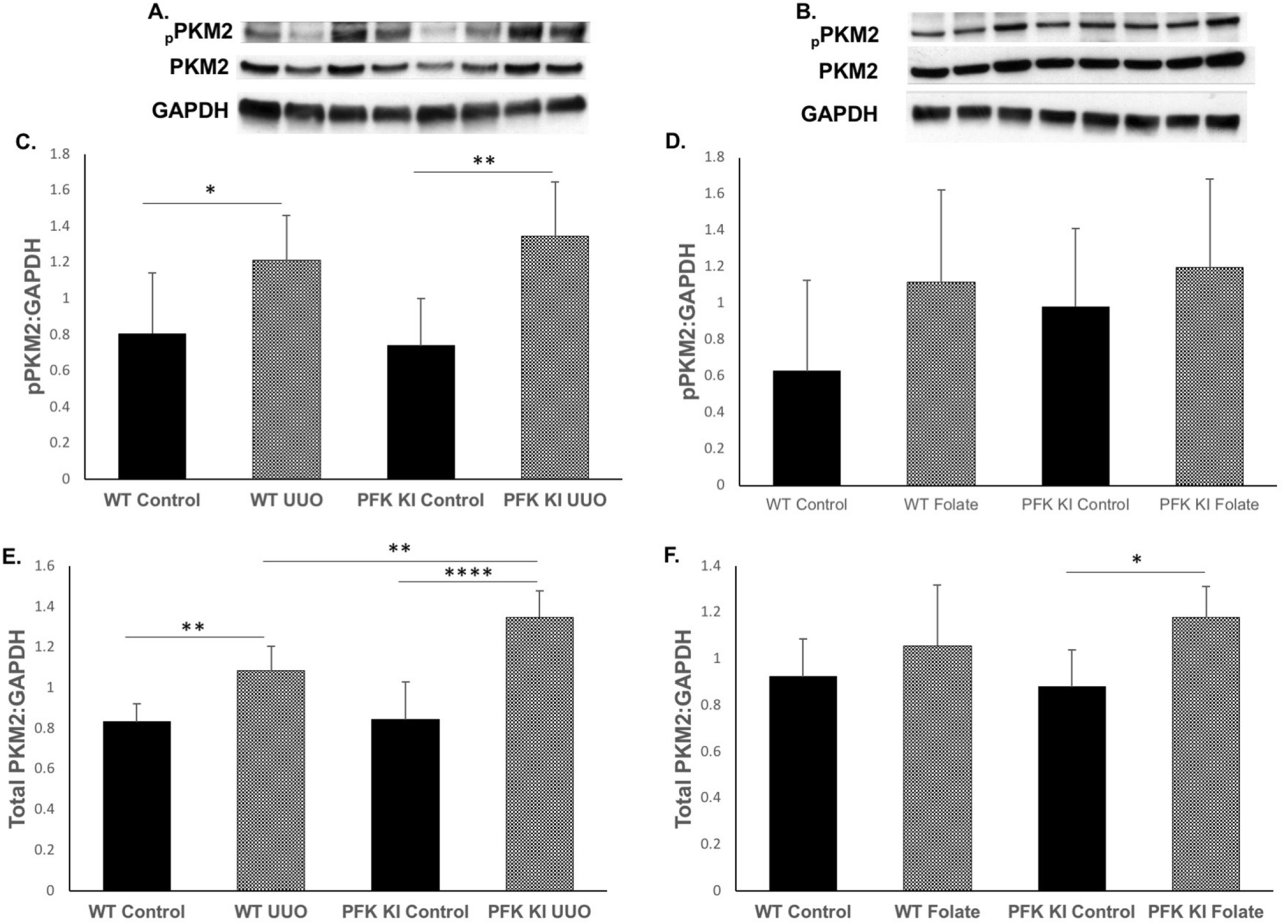
PKM2 and pPKM2 expression in WT mice and PFKFB2 KI mice with UUO and FAN. (**A**,**C**,**E**) Representative Western blots showing significantly increased expression of PKM2 and pPKM2 in WT and PFKFB2 KI mice with UUO, as determined by densitometry. There was a significant difference in expression of PKM2 but not pPKM2 between WT and PFKFB2 KI mice. (**B**,**D**,**F**) Representative Western blots showing no change in expression of PKM2 and pPKM2 in WT with FAN, as determined by densitometry. There was a difference in the PFKFB2 KI mice between control and FAN mice, but no difference when compared to WT mice. n = 6 mice per panel. *P < 0.05, **P < 0.01, ***P < 0.001. Mean + S.D.

In view of evidence that pPKM2 and PKM2 are increased in UUO^5^, we performed Western blots in the UUO and FAN models (Fig. 6A,B). Both pPKM2 and PKM2 were strongly increased in the UUO model in WT and PFKFB2 KI mice, and there was a significant difference in PKM2 expression between WT and PFKFB2 KI mice with UUO (Fig. 6C,E). Interestingly, there was no difference between WT mice with FAN compared to controls, and no difference between WT and PFKFB2 KI mice with FAN (Fig. 6D,F).

### Renal fibrosis in disease models

**Figure 7:**
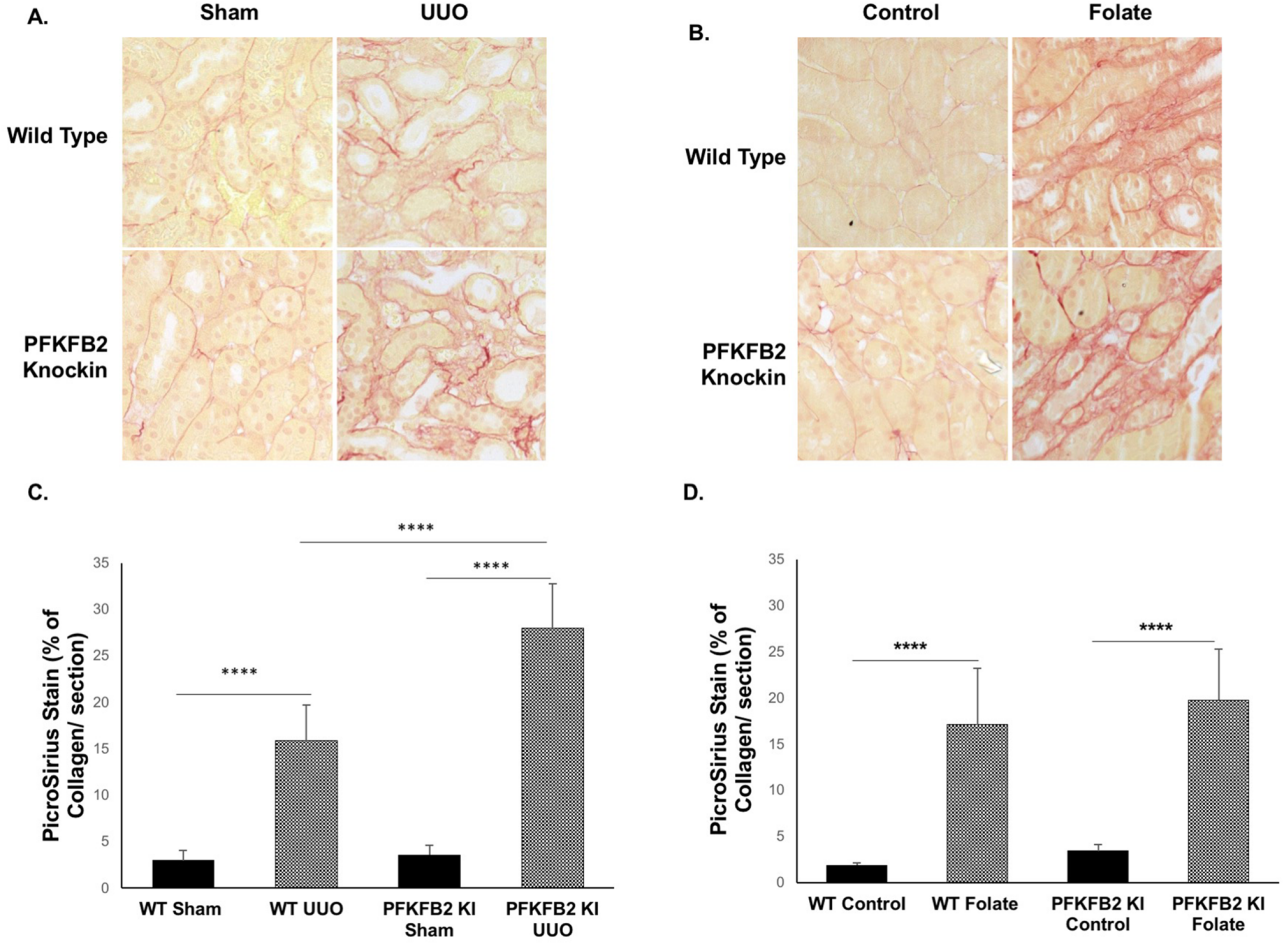
Histochemical evaluation of renal fibrosis in WT and PFKFB2 KI mice with UUO and FAN. Representative images of Picro-Sirius Red stained kidney sections from WT and PFKFB2 KI mice with UUO (**A**) and FAN (**B**). Quantification of Picro-Sirius Red stained kidney sections showing increased staining in PFKFB2 KI mice with UUO compared to WT mice (**C**). There was no difference between WT and PFKFB2 KI mice with FAN (**D**). (n = 7 for all groups). ****P < 0.0001. Mean + S.D.

**Figure 8:**
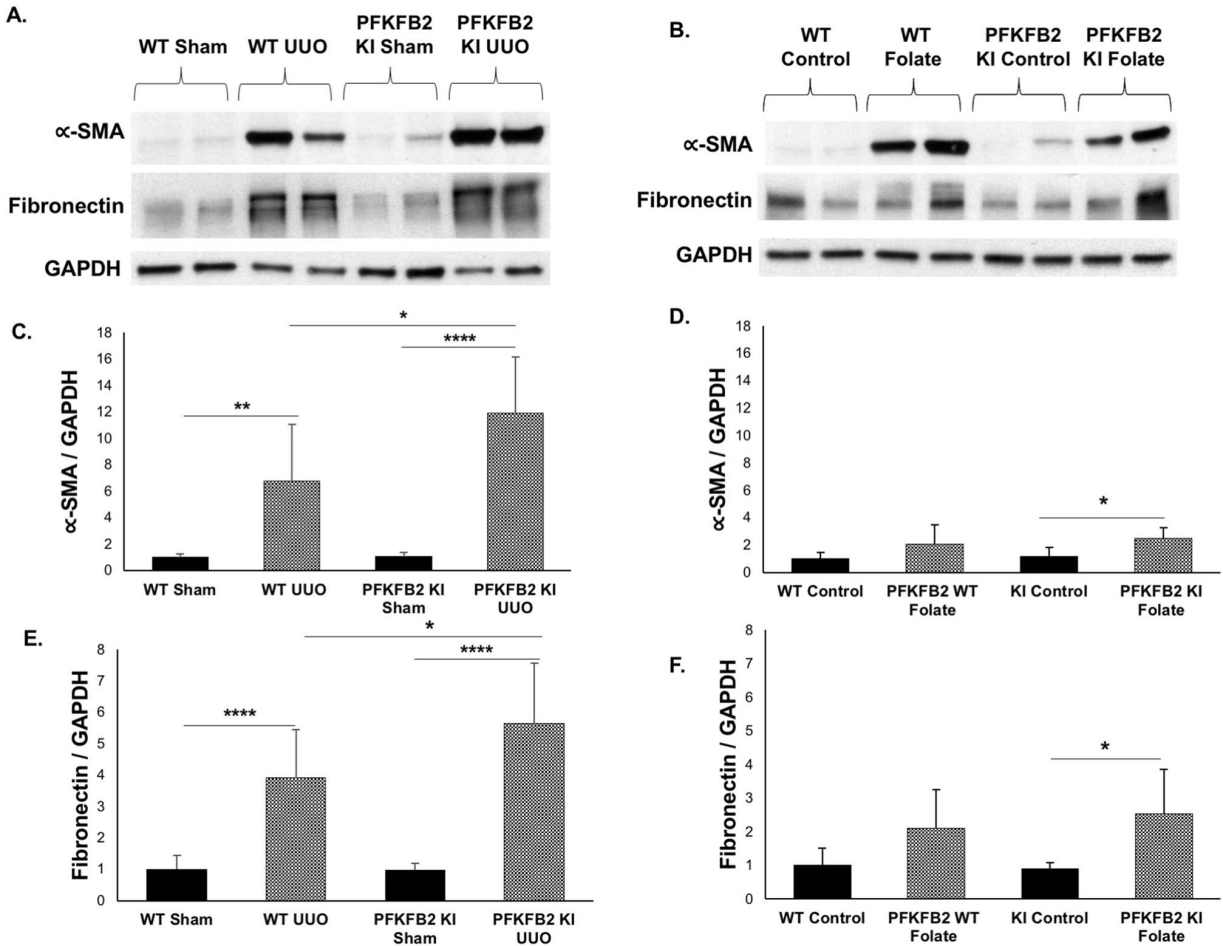
Expression of α-SMA and fibronectin protein in WT and PFKFB2 KI mice with UUO and FAN. Representative Western blots are shown for the UUO (**A**) and FAN (**B**) models. Densitometric analysis shows an increase in α-SMA (**C**) and fibronectin (**E**) in the UUO model in the PFKFB2 KI mice compared with WT, but no difference in the FAN model between these groups (**D**, **F**). Mean + S.D. n = 6 per group.

**Figure 9:**
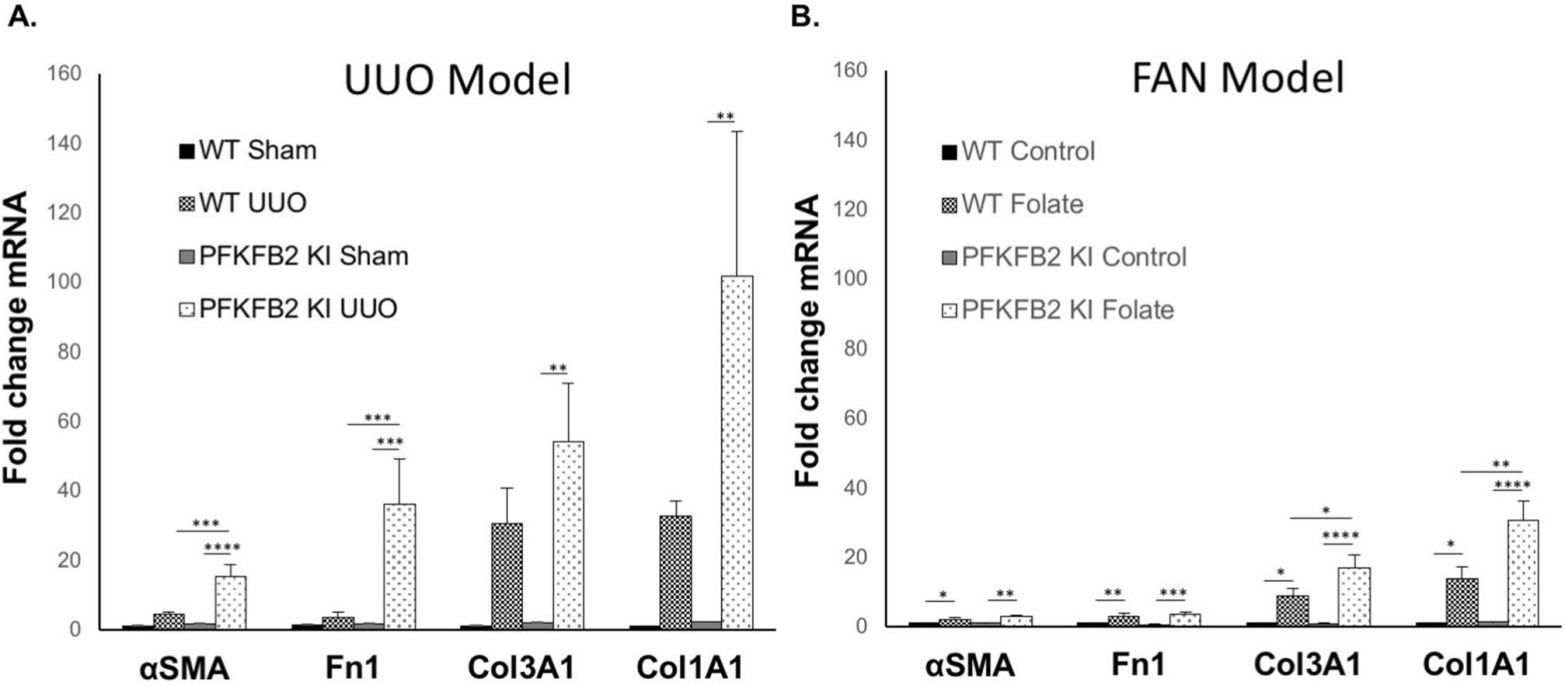
mRNA expression of extracellular matrix proteins in WT and PFKFB2 KI mice with UUO and FAN. α-SMA, Fibronectin, Collagen3α1 and Collagen1α1 were assessed using qRT-PCR in the UUO (**A**) and FAN (**B**) models. Quantitative qRT-PCR data were normalized to GAPDH and expressed as the fold-change compared with WT control. mRNA expression for α-SMA and fibronectin increased significantly in the PFKFB2 KI mice with UUO compared to WT (**A**), but not in the FAN model (**B**). In contrast, Collagen3α1 and Collagen1α1 increased significantly in the PFKFB2 KI mice with FAN compared to WT (**B**), but not in the UUO model (**A**). Mean + S.E.M. n = 7 per group. *P < 0.05, **p < 0.01, ***p < 0.001, ****P < 0.0001.

In the UUO model, there was a significant increase in fibrosis, assessed by staining of sections with Picrosirius Red in the PFKFB2 KI compared to WT mice (Fig. 7A, C). This was associated with increases in α-SMA and fibronectin the PFKFB2 KI compared to WT mice by Western blot (Fig. 8A,C,E). This was confirmed by qRT-PCR, which showed increased fibronectin and α-SMA in the PFKFB2 KI compared to WT mice (Fig. 9A). Values for collagen 3A1 and 1A1mRNA were very variable in this model, and there was no significant difference between WT and PFKFB2 KI mice with renal fibrosis (Fig. 9A).

In the FAN model, by contrast, there was no difference in staining for Picrosirius Red in the PFKFB2 KI mice compared to WT (Fig. 7B,D), and there was also no difference in expression of α-SMA or fibronectin by Western blot (Fig. 8B,D,F). Despite the absence of any significant change in expression of fibronectin and α-SMA protein, qRT-PCR showed an increased expression of mRNA species for col3A1 and col1A1 in the PFKFB2 KI mice with FAN compared to WT mice with FAN (Fig. 9B). Overall, this indicated a marginal effect of the PFKFB2 KI mutation on renal fibrosis in the FAN model.

To determine whether the change in fibrotic markers was associated with greater inflammation, possibly as a causative factor, tissues were probed by qRT-PCR for expression of F4-80, MCP-1 and Mac-2. However, there was no difference in macrophage associated genes in the PFKFB2 mice compared to WT in either model (Supplementary Fig. 2).

### Glycogen accumulation in kidneys of mice with UUO and FAN

**Figure 10:**
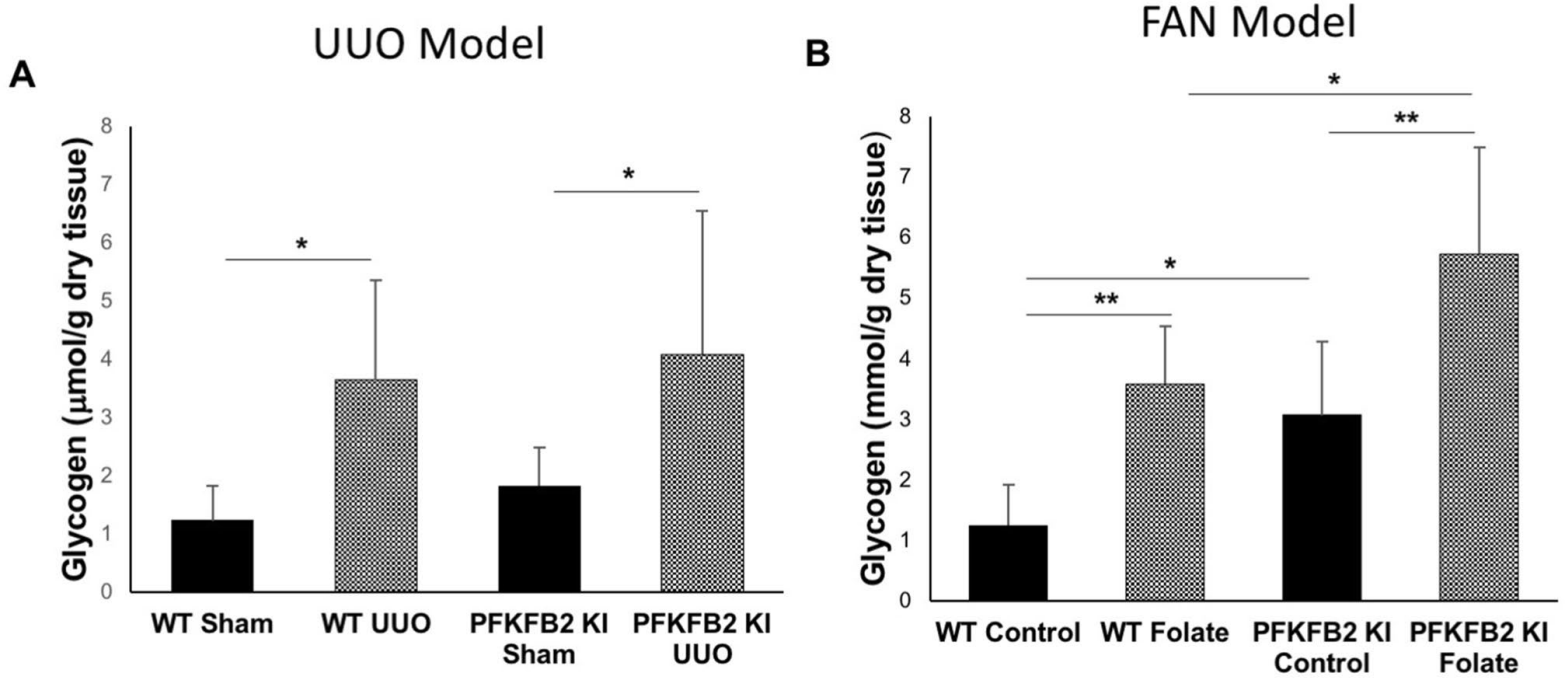
Glycogen accumulation in WT and PFKFB2 KI mice with UUO and FAN. Glycogen increased in both UUO (**A**) and FAN (**B**) models in WT and PFKFB2 KI mice. In the FAN model, there was a significant increase in glycogen in the untreated PFKFB2 KI control mice compared to WT and a non-significant trend in the sham-operated UUO mice. In the FAN model, there was a significantly greater increase in PFKFB2 mice with disease compared with WT (**B**). This did not occur in the UUO mice (**A**). *P < 0.05, **P < 0.01, ***P < 0.001. Mean + S.D. n = 7 per group.

We proposed that glycogen storage by the kidney might have been affected in mice with a block in glycolysis. Glycogen accumulation was assessed by direct measurement. In the UUO model, there was an increase in both PFKFB2 KI and WT mice following UUO, but no difference between the two (Fig. 10A). In the FAN model there was also an increase in both strains of mice but, in contrast to the mice with UUO, it was greater in the PFKFB2 KI mice (Fig. 10B**)**.

## Discussion

To determine the role of glycolysis in renal fibrosis, we generated a transgenic mouse with mutations in the regulatory phosphorylation sites of PFKFB2. When phosphorylated by a number of protein kinases, PFKFB2 increases synthesis of fructose-2,6-bisphosphate, a strong activator of PFK1, the major control point in glycolysis^12, 14^. It would be predicted, therefore, that inactivating mutation of these phosphosites in PFKFB2 would reduce the ability of cells to increase glycolysis in response to stimulation. This was confirmed in cultured TEC from PFKFB2 KI mice, which had a reduced ability to increase glycolysis following a glucose stimulus.

PFKFB2 is highly expressed in the heart and kidney^17^, and has sometimes been described as the kidney isoform of PFK-2/FBPase-2^15^. We were interested to determine its likely distribution in the kidney in renal fibrosis, since this would affect our interpretation of studies performed in the PFKFB2 KI mice. Western blots performed using cultured TEC and fibroblasts from WT mice showed that expression of PFKFB2 was only detectable in TEC. This was confirmed by immunohistochemical staining showing expression predominantly in renal tubules, especially distal. This data suggests that any effect of PFKFB2 KI mutation on renal fibrosis is likely to represent an effect in renal tubular cells rather than fibroblasts.

To determine the effect of reduced regulation of glycolysis on the development of renal fibrosis, we utilized two models of renal fibrosis. Both models affect the renal tubule primarily but we assumed that the UUO model, which is reliant on pressure transduced from the ureter might have a greater effect on the distal tubule than the FAN model, which originates from folate filtered by the glomerulus and precipitating in renal tubules^26^. The FAN model is thought to produce both direct pan-tubular toxicity as well as crystal obstruction and is a more mild overall injury^26^. The proximal renal tubule is dependent on fatty acid oxidation for energy generation, whilst the distal tubule relies more heavily on glycolysis^27^. The data showed significantly increased fibrosis in the UUO model, whereas there was only a marginal effect in the FAN model.

This study demonstrates that glycogen accumulates in fibrotic kidneys. We are not aware of this observation having been made before. Glycogen accumulation in the kidney is well described in diabetes and glycogen storage disease, and there is some evidence that it causes renal fibrosis^28^. In this study, it was not increased in the PFKFB2 KI mice with UUO compared to WT, so is unlikely to have contributed to the marked difference seen in fibrosis. Another unexplained finding was the absence of any difference in glycogen storage between wild type and PFKFB2 KI mice. The latter are presumed to have a defect in glycolysis so there might be more glucose available for glycogen synthesis.

Some renal diseases have been characterized by aerobic glycolysis^5, 29–32^, or the Warburg effect, where, despite the presence of oxygen, pyruvate generated from glucose is metabolized to lactate in the cytosol, rather than entering mitochondria for oxidative metabolism. This has been noted in autosomal dominant polycystic kidney disease and diabetic nephropathy^29–31^. There have been several studies indicating that this also occurs in the UUO model of renal fibrosis. Ding et al. showed that 2-deoxyglucose, which inhibits the activity of hexokinase at the beginning of the glycolytic pathway, reduced fibrosis in the UUO model, but probably due to an effect on fibroblasts rather than tubular cells^5^. Wei et al. used inhibitors of PKM2 and PDK, at the end of the glycolytic pathway, to reduce fibrosis in the UUO model^6^. Once again, they attributed this predominantly to an effect on fibrobasts. Finally, Zhou et al. reported that inactivation of PKM2 in mouse proximal tubular cells reduced fibrosis in the UUO model^7^. In the present study, by contrast, the data suggest that reducing glycolysis predominantly in distal tubular epithelial cells does not have a beneficial effect.

A confusing finding in the current study is that two enzymes that affect the rate of glycolysis responded differently in the UUO model. PKM2 increased, which would be expected to increase glycolysis, while the reduction in PFKFB2 expression and phosphorylation should decrease it. There are two potential explanations. The first is that Limbutara et al., in their recent paper, have reported that PKM2 and PFKFB2 have different protein distributions in the rat renal tubules^33^. Assuming that mouse and rat are similar, then PKM2 is predominantly found in the outer and inner medullary collecting duct and cortical collecting duct while PFKFB2 is found in parts of the proximal tubule as well as the connecting tubule and cortical collecting duct. The only segment with significant overlap of the two seems to be in the cortical collecting duct. Secondly, PKM2 tetramers are responsible for the increase in aerobic glycolysis and we do not know if the increase in PKM2 protein leads to more tetramers^34^. The predicted decrease in F-1,6-P2 in PFKFB2 KI mice would decrease the ability of PKM2 to form tetramers, and so increase the later stages of glycolysis^34^.

In summary, this study has demonstrated that inhibition of glycolysis in the UUO model has a marked effect on the development of renal fibrosis. The FAN model was much less affected, probably due to the greater location of injury in proximal tubules that use fatty acid predominantly and glycolysis is a relatively minor energy source compared with distal tubules. In distal tubles, glycolysis is a more important source of energy and PFKFB2 is more strongly expressed. These studies suggest that strategies relying on inhibition of glycolysis to improve renal fibrosis must take into account the type and location of the cells being inhibited.

## Supplementary information

Supplementary file 1
