# Supplementary material for: Mutation of regulatory phosphorylation sites in PFKFB2 worsens renal fibrosis
Source: Sci Rep. 2020 Sep 3;10:14531. doi: 10.1038/s41598-020-71475-z (PMC7471692; doi:10.1038/s41598-020-71475-z)
Supplement: Supplementary file 1 [file 41598_2020_71475_MOESM1_ESM.pdf]

MUTATION OF REGULATORY PHOSPHORYLATION SITES IN PFKFB2 WORSENS RENAL FIBROSIS

MARDIANA LEE, GEOFF HARLEY, MARINA KATERELOS, KURT GLEICH, MITCHELL A. SULLIVAN, ADRIENNE LASKOWSKI, MELINDA COUGHLAN, SCOTT A. FRASER, PETER F. MOUNT, DAVID A. POWER.

Supplementary Figures and Tables

Page 2: Supplementary Figure 1. PFKFB2 KI mice gene targeting strategy.

Page 3: Supplementary Figure 2. Macrophage accumulation in WT and PFKFB2 KI mice with UUO and FAN.

Page 4: Supplementary Table 1. Primer sequences for qRT-PCR.

Page 5: Supplementary Figure 3a. Full-length Western blot images for Fig 2A.

Page 6: Supplementary Figure 3b. Full-length Western blot images for Fig 2D.

Page 7: Supplementary Figure 4. Full-length Western blot images for Fig 4A.

Page 8: Supplementary Figure 5a. Full-length Western blot images for Fig 5A.

Page 9: Supplementary Figure 5b. Full-length Western blot images for Fig 5D.

Page 10: Supplementary Figure 6a. Full-length Western blot images for Fig 6A.

Page 11: Supplementary Figure 6b. Full-length Western blot images for Fig 6B.

Page 12: Supplementary Figure 7a. Full-length Western blot images for Fig 8A.

Page 13: Supplementary Figure 7b. Full-length Western blot images for Fig 8D.

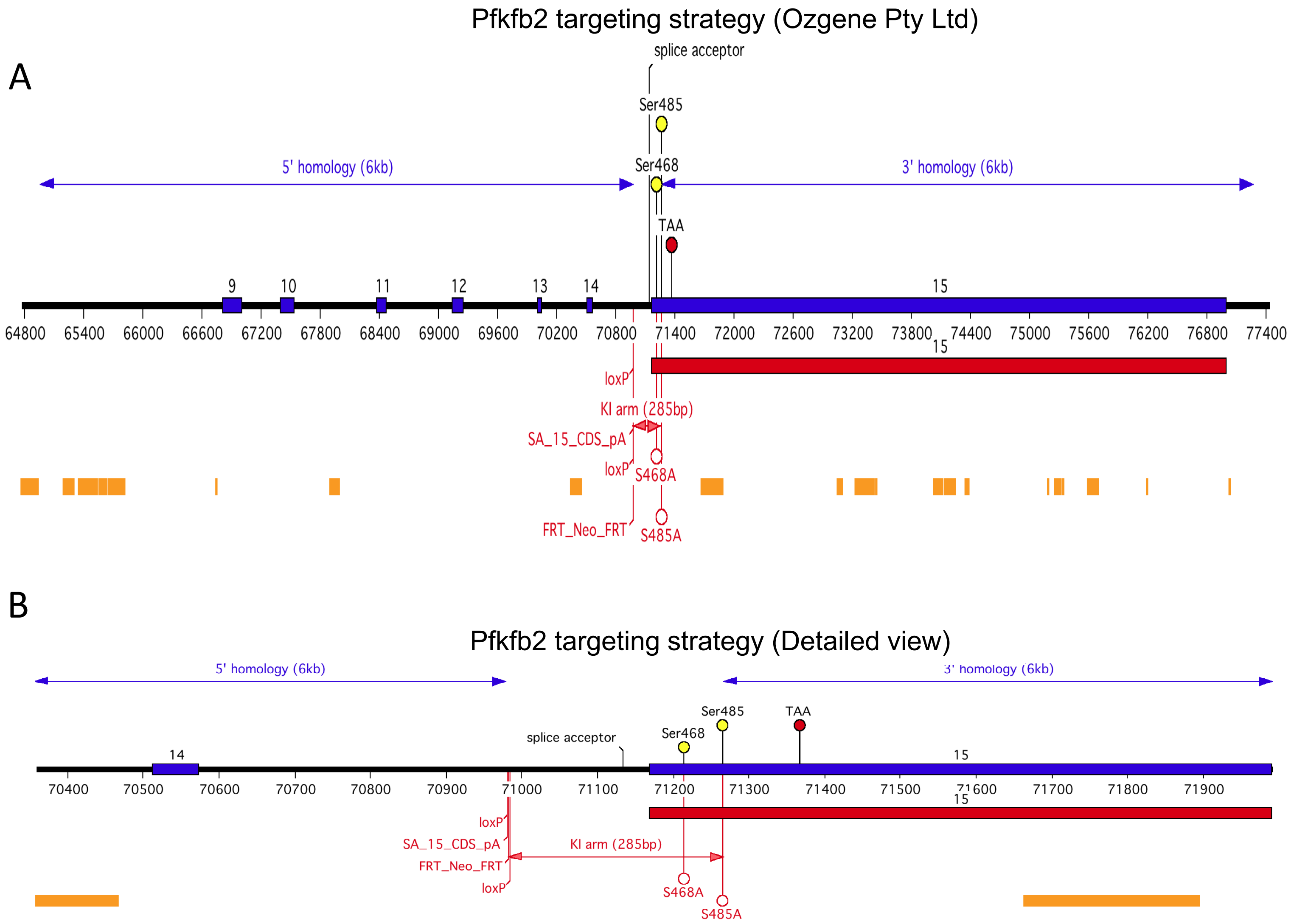

Supplementary Figure 1. PFKFB2 KI mice gene targeting strategy. A: Overview; B: Detail. Exon 15 is highlighted by a red bar. Red lines indicate insertion of targeting features. A floxed copy of exon 15's wild-type coding sequence (SA_15_CDS), followed by a polyadenylation signal (pA) was inserted into intron 14-15. The floxed segment included the neomycin marker (Neo), itself flanked by FRT sites. The Ser to Ala mutations were targeted into exon 15. The targeting vector homology arms are indicated by double blue arrows (not shown to scale in detail view). Figure provided by Ozgene Pty Ltd.

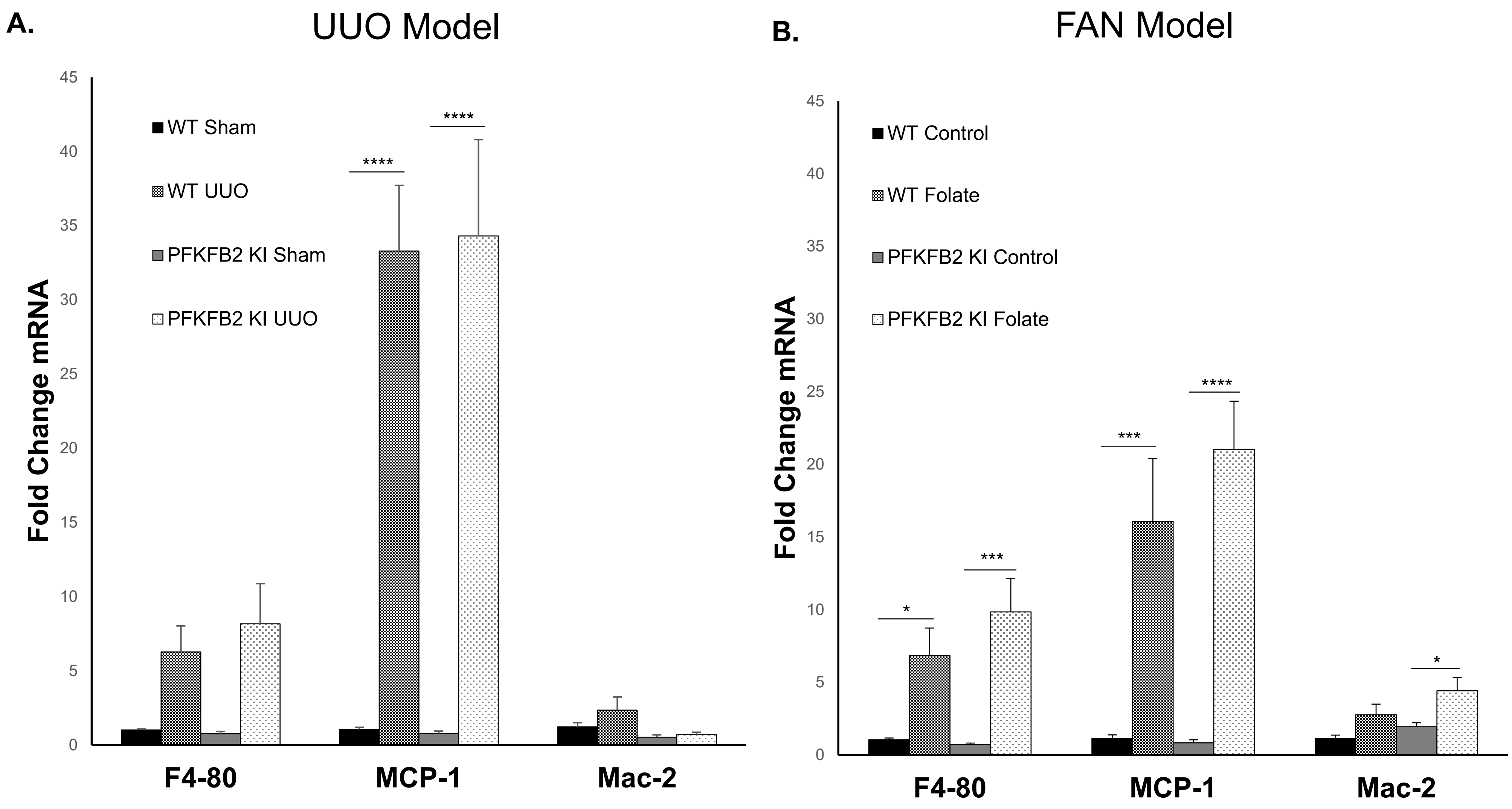

Supplementary Figure 2. Macrophage accumulation in WT and PFKFB2 KI mice with UUO and FAN. (A) RT-PCR analysis showing increased mRNA expression of F4-80, MCP-1 and Mac-2 in the UUO (A) and FAN (B) models. Although macrophages increased in both models, there was no difference between WT and PFKFB2 KI groups. * $P<0.05$, **$P<0.01$, *** $P<0.001$, ****$p<0.0001$. Mean + S.E.M. n=7 per group.

Supplementary Table 1. Primer sequences for qRT-PCR

Gene Name	Forward Primer	Reverse Primer
α -SMA	CAGGCATGGATGGCATCAATCAC	ACTCTAGCTGTGAAGTCAGTGTCG
Collagen 1A1	TGACTGGAAGAGCGGAGAGT	GTTCGGGCTGATGTACCAG
Collagen 3A1	ACAGCTGGTGAACCTGGAAG	ACCAGGAGATCCATCTCGAC
Fibronectin	CGAGGTGACAGAGACCACAA	CTGGAGTCAAGCCAGACACA
Cpt1	GGTCTTCTCGGGTCGAAAGC	TCCTCCCACCAGTCACTCAC
ACox	CTTGGATGGTAGTCCGGAGA	TGGCTTCGAGTGAGGAAGTT
GAPDH	AAGGTCATCCCAGAGCTGAA	CTGCTTCACCACCTTCTTGA
F4/80	TCCTGCTGTGTCGTGCTGTTC	GCCGTCTGGTTGTCAGTCTTGTC
MCP-1	CAAGAAGGAATGGGTCCAGA	GTGCTGAAGACCTTAGGGCA
Mac-2	GCTTATCCTGGCTCAACTG	TTCACTGTGGCCCATGATTGT
Hk(Hex)	GGAGCAGTGGACCAGGGTA	GAAGTTCAGCTGTTTTTGAATTG
Glut1	ACACTCACCACGCTTTGGTC	ACACACCGATGATGAAGCGG
Pfk1	CCATGTTGTGGGTGTCTGAG	ACAGGCTGAGTCTGGAGCAT

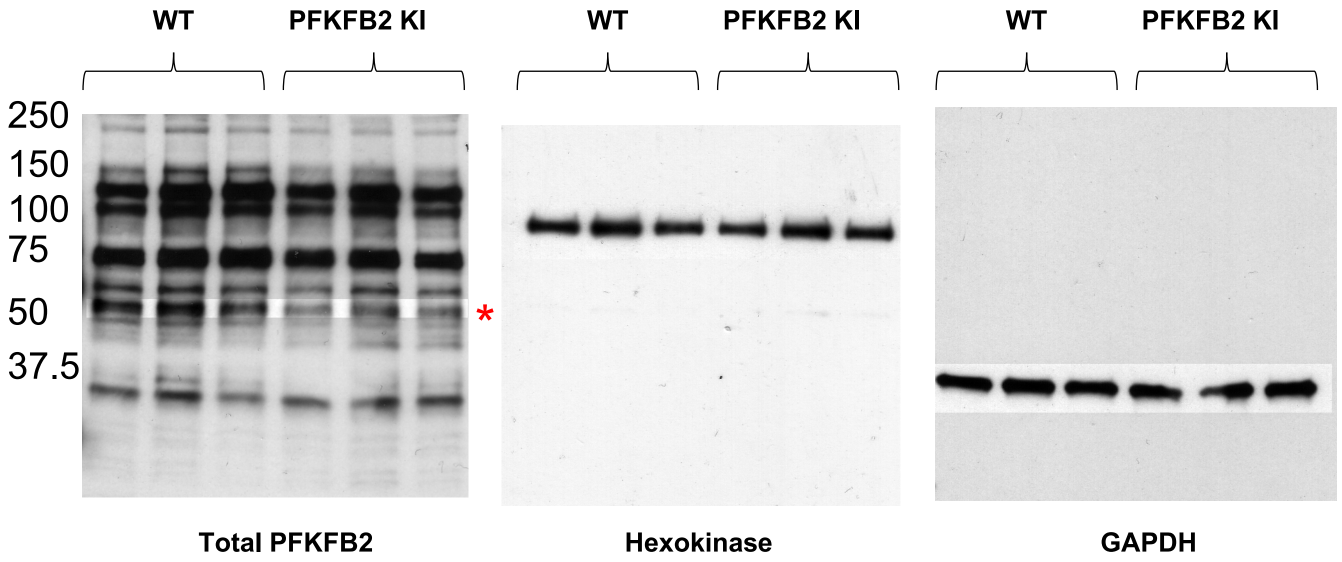

Supplementary Figure 3a: Full-length Western blot images for PFKFB2 (red asterisk denotes PFKFB2 band), Hexokinase and GAPDH representing Western blot images in Figure 2A.

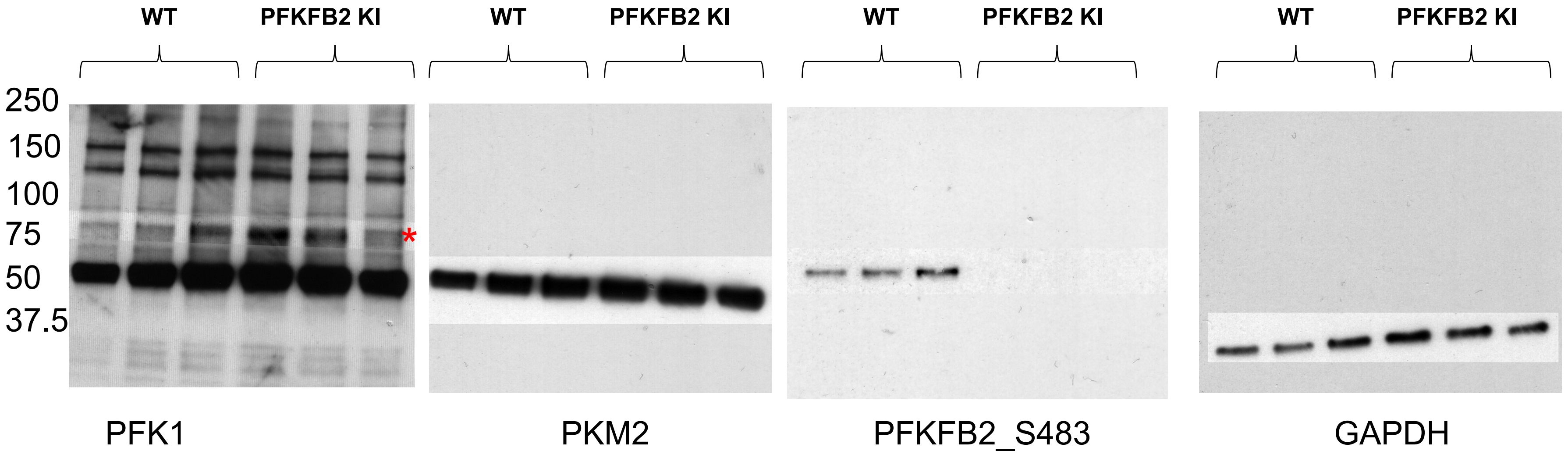

Supplementary Figure 3b: Full-length Western blot images for PFK1 (red asterisk denotes PFK1 band), PKM2, p-PFKFB2 and GAPDH representing Western blot images in Figure 2D.

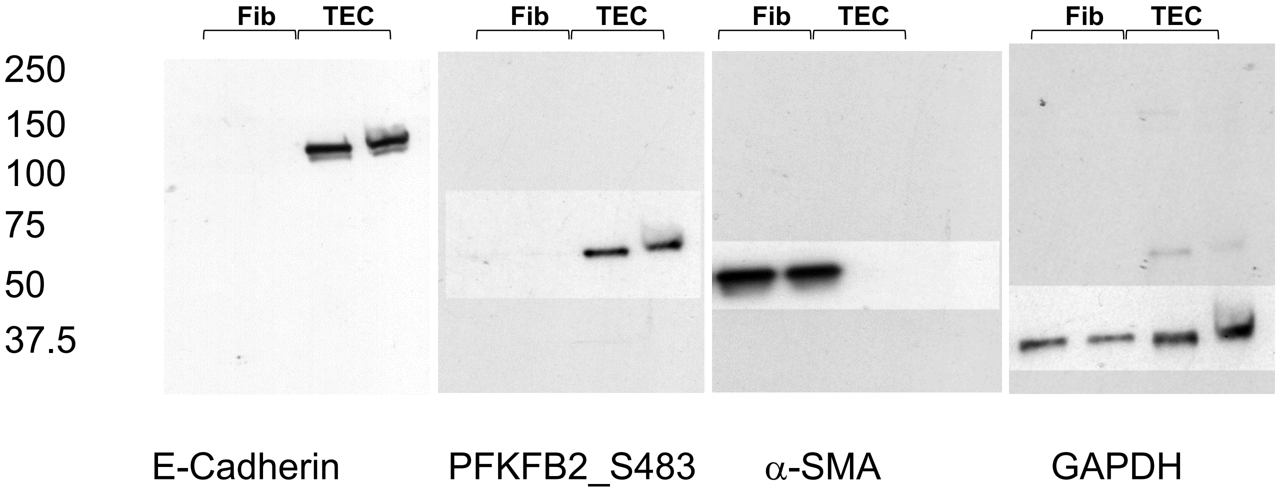

Supplementary Figure 4: Full-length Western blot images for E-Cadherin, p-PFKFB2, α-SMA and GAPDH representing Western blot images in Figure 4A.

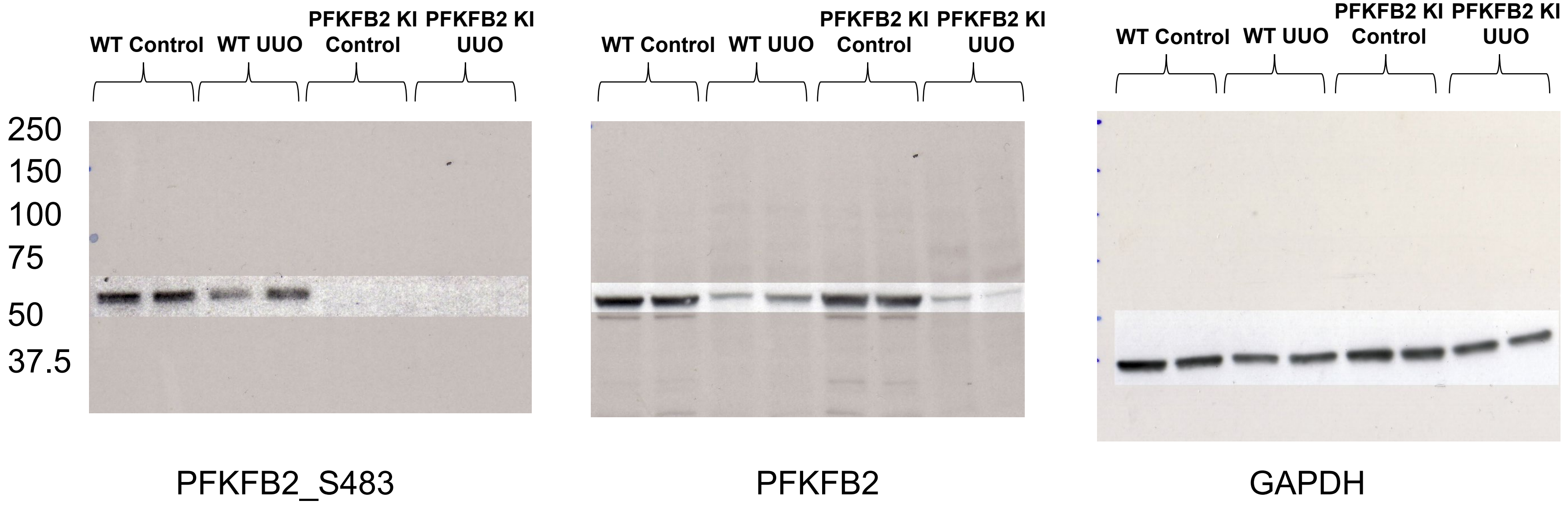

Supplementary Figure 5a: Full-length Western blot images for p-PFKFB2, PFKFB2 and GAPDH representing Western blot images in Figure 5A.

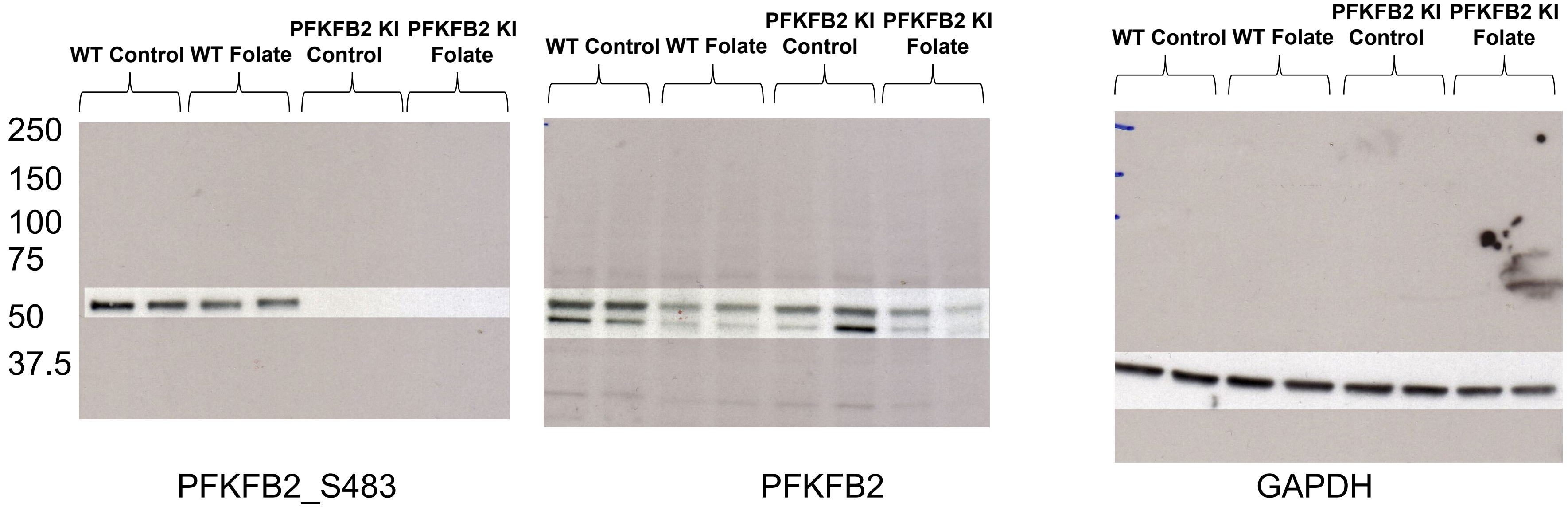

Supplementary Figure 5b: Full-length Western blot images for p-PFKFB2, PFKFB2 and GAPDH representing Western blot images in Figure 5D.

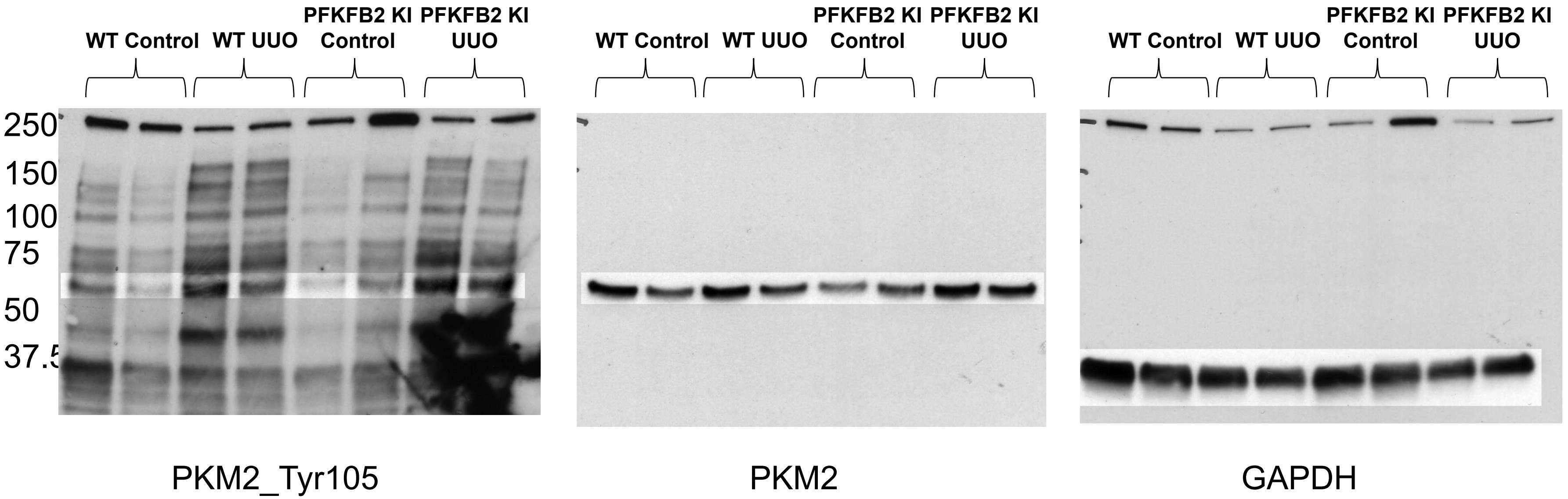

Supplementary Figure 6a: Full-length Western blot images for p-PKM2, PKM2and GAPDH representing Western blot images in Figure 6A.

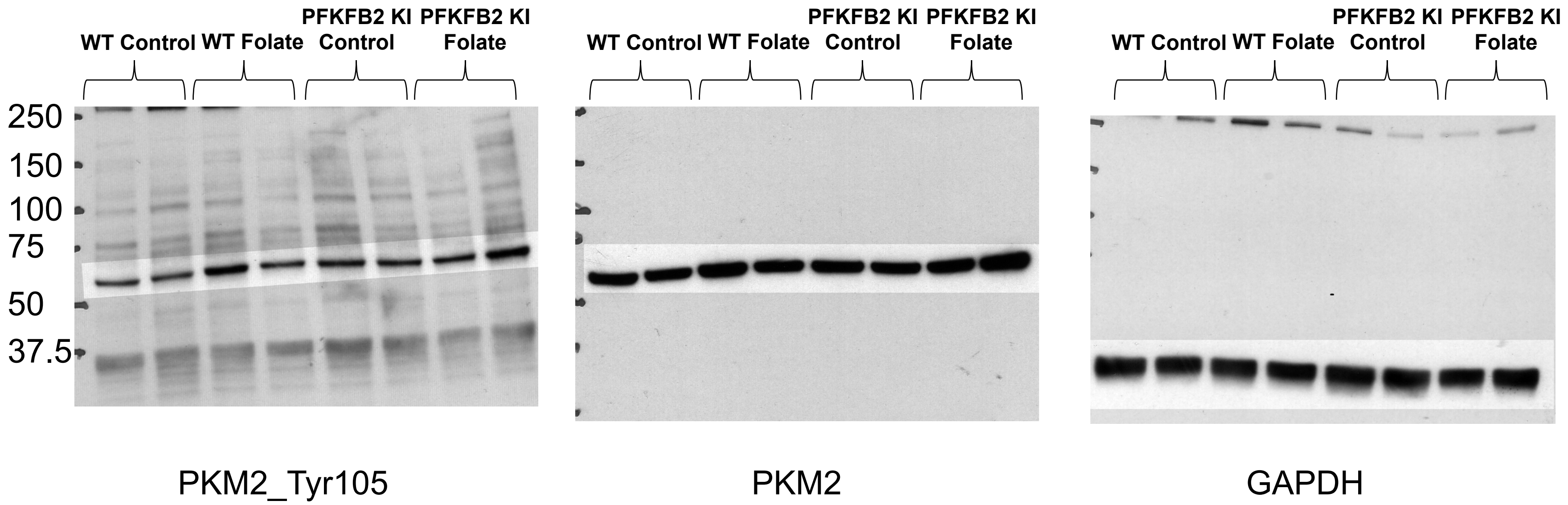

Supplementary Figure 6b: Full-length Western blot images for p-PKM2, PKM2 and GAPDH representing Western blot images in Figure 6B.

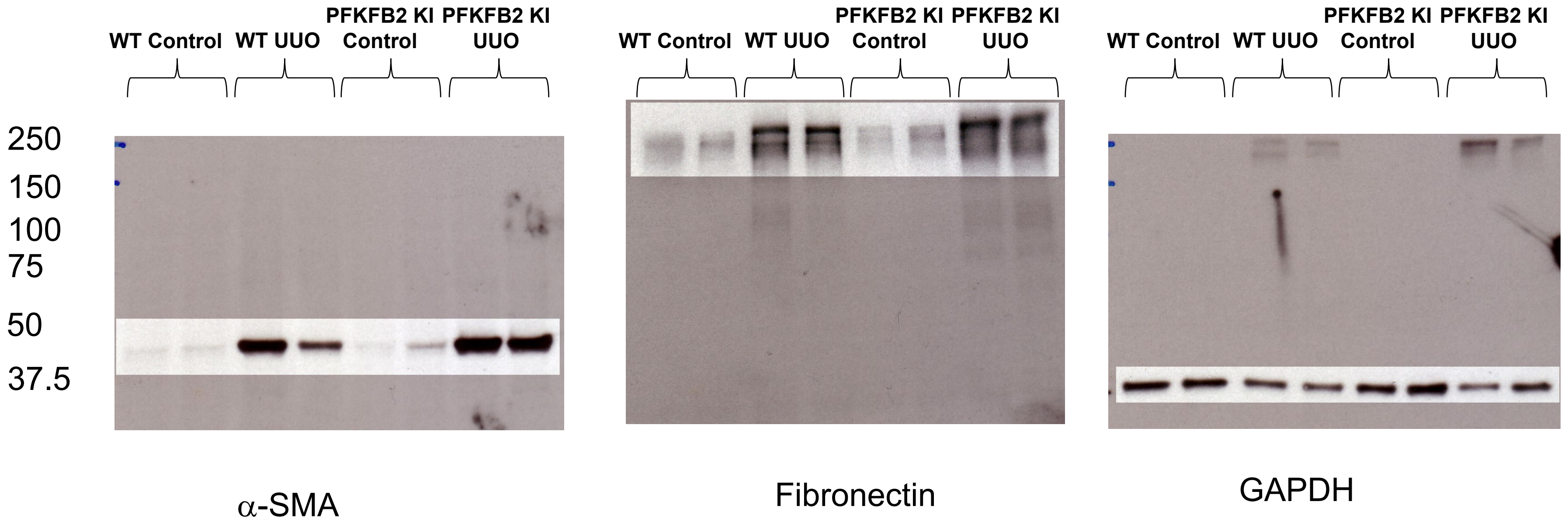

Supplementary Figure 7a: Full-length Western blot images for α-SMA, Fibronectin and GAPDH representing Western blot images in Figure 8A.

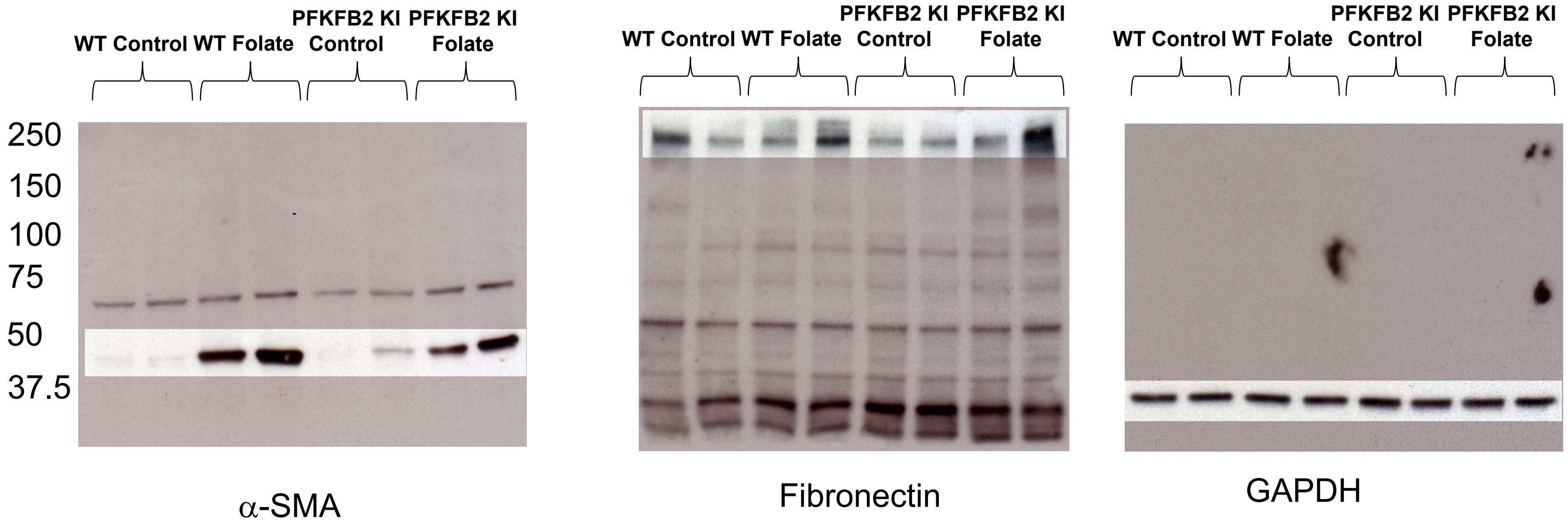

Supplementary Figure 7b Full-length Western blot images for α-SMA, Fibronectin and GAPDH representing Western blot images in Figure 8B.
